# MiR-501-3p/SPC24 axis affects cell proliferation, migration, invasion, apoptosis, and prognosis in renal cell carcinoma

**DOI:** 10.1590/1414-431X2024e13507

**Published:** 2025-02-03

**Authors:** Aidi Liang, Jiapeng Huang, Xinyi He, Xinru Tang, Xuncan Xu, Ming Chen, Lei Meng, Canbin Lin

**Affiliations:** 1 Department of Urology, The First Affiliated Hospital of Guangzhou University of Chinese Medicine, Guangzhou, Guangdong, China; 2 Guangdong Clinical Research Academy of Chinese Medicine, Guangzhou, Guangdong, China; 3 The First Clinical Medical School of Guangzhou University of Chinese Medicine, Guangzhou, Guangdong, China; 4 Department of Traditional Chinese Medicine, Jinan University, Guangzhou, Guangdong, China; 5 Guangdong Provincial Key Laboratory of Traditional Chinese Medicine, Guangzhou, Guangdong, China

**Keywords:** MiR-501-3p, SPC24, Renal cell carcinoma, ACHN, 786-O

## Abstract

It has been confirmed that the expression of miR-501-3p is closely related to the behavior of several cancers. This study aimed to elucidate the effects of miR-501-3p/SPC24 axis on the behavior of renal cancer cells and to identify its prognostic value in renal cancer. First, the expression of miR-501-3p in the renal cell carcinoma (RCC) cell line was detected using real-time quantitative polymerase chain reaction (RT-qPCR). Second, cell function identification experiments were performed, including CCK-8, scratch, transwell invasion, and flow cytometry assays. Several databases were applied to explore the possible mechanism of miR-501-3p tumor suppressor effect in RCC. To explore the value of miR-501-3p/SPC24 axis in predicting renal cancer patient overall survival (OS), GEPIA (http://gepia.cancer-pku.cn/index.html) was used. Finally, western blot was performed to detect the expression level of SPC24 in renal cancer cells predicted by bioinformatics analysis. Dual-Luciferase Reporter Assay was used to verify if SPC24 is a target of mir-501-3p. MiR-501-3p was found to be down-regulated in cancer cells and tissues and to play a role in suppressing tumor cell proliferation, cell viability, cell migration, and cell invasion, while promoting apoptosis. We also found that high expression levels of SPC24 were associated with shorter OS time in patients diagnosed with renal cell carcinoma. In addition, the results of TCGA data analysis and western blot showed that the tumor suppressor effect of miR-501-3p may be achieved by targeting SPC24. The MiR-501-3p/SPC24 axis affects cell proliferation, migration, invasion, apoptosis, and prognosis in renal cell carcinoma.

## Introduction

Renal cell carcinoma (RCC) is a malignant tumor originating in the renal tubular epithelium. According to the data of 2020 Global Cancer Statistics (1), there were 431,288 new cases of renal cell carcinoma worldwide in 2020, accounting for 2.2% of all malignant tumors. In addition, there were 175,098 deaths from kidney cancer, accounting for 1.8% of all cancer sites. RCC is not sensitive to radiation therapy or chemotherapy (2). The main treatment method at the moment is surgery (3). The pathogenesis of kidney cancer is currently unknown. Many of the kidney cancer cases are advanced when diagnosed, and the loss of surgical opportunities leads to poor prognosis (4). Therefore, it is important to identify and study molecular markers related to kidney cancer.

MicroRNAs are non-coding RNAs with a length of about 22 nucleotides (5). They act on messenger RNA (mRNA) after gene transcription to indirectly regulate gene expression, thereby affecting cellular behavior (6,7). In recent years, many scholars found that miR-501-3p is dysregulated in different tumor tissues and have performed a series of studies, confirming that miR-501-3p is related to the occurrence and development of liver cancer, prostate cancer, lung cancer, and colon cancer (8-11). SPC24, an important component of the NDC80 complex, plays an important role in the coupling of centromere to spindle microtubules and in the precise separation of chromosomes during mitosis. It was discovered that SPC24 is abnormally expressed in a variety of diseases including neoplastic diseases, and SPC24 may act as an oncogene in kidney cancer via regulation of SRY-box transcription factor 2 (12).

However, the upstream regulatory mechanism of SPC24 remains unclear. In addition, the relationship between miR-501-3p and SPC24 still needs to be uncovered in kidney cancer. In this study, bioinformatics analysis revealed that mir-501-3p was correlated with SPC24, and the upstream regulatory molecule of SPC24 might be mir-501-3p. Therefore, we hypothesized that mir-501-3p plays a role in renal cancer via SPC24. In order to verify this hypothesis, we chose the 786-O cell line of primary clear cell carcinoma (the most frequent subtype) from a 58-year-old male and the ACHN cell line of a metastatic cancer from a 22-year-old male to investigate the role of mir-501-3p in renal cancer and the relationship between mir-501-3p and SPC24.

## Material and Methods

### Cell culture and transfection

The human ACHN and 786-O cell lines were obtained from the American Type Culture Collection (ATCC, USA). RCC cells were cultured in basic Dulbecco's modified Eagle medium (DMEM) (Gibco; USA) or Roswell Park Memorial Institute 1640 medium (Gibco) containing 10% fetal bovine serum (FBS; Gibco), 1% PS (100 μL/mL penicillin and 100 mg/mL streptomycin sulfates; Gibco), and 1% glutamax (Gibco). Cells were incubated at 37°C in a 5% CO_2_ incubator. Subsequently, transfections were performed in RCC cell lines to either enhance or suppress miR-501-3p mRNA levels. Opti-MEM^®^ I Reduced Serum Medium (Gibco; Thermo Fisher Scientific, Inc., USA) mixed with lipofectamine 2000 (Invitrogen, USA) and a miR-501-3p mimic or inhibitor (Shanghai GenePharma Co., Ltd., China) was used for transfections. Furthermore, RT-qPCR was performed to validate transfection efficiency. The primer sequences of miR-501-3p mimic and inhibitor are shown in Table 1.

**Table 1:** Sequences of primers and microRNAs.

Primer/microRNA	Sequence
miR-501-3p	Forward: 5'-AATGCACCCGGGCAAGGATTCT-3′
	Reverse: Universal primers (miScript SYBR Green PCR kit)
U6	Forward: 5'-CTCGCTTCGGCAGCACA-3′
	Reverse: 5'-ACGCTTCACGAATTTGCGT-3′
miR-501-3p mimic	Forward: 5'-AAUGCACCCGGGCAAGGAUUCU-3′
	Reverse: 5'-AAUCCUUGCCCGGGUGCAUUUU-3′
Mimic nc	Forward: 5'-UUGUACUACACAAAAGUACUG-3′
	Reverse: 5'-GUACUUUUGUGUAGUACAAUU-3′
miR-501-3p inhibitor	5'-AGAAUCCUUGCCCGGGUGCAUU-3′
Inhibitor nc	5'-CAGUACUUUUGUGUAGUACAA-3′

miR: microRNA; nc: negative control;PCR; polymerase chain reaction.

### RNA extraction and cDNA synthesis

TRIzol (Invitrogen; Thermo Fisher Scientific, Inc.) was used to isolate total RNA from renal cancer cells. Extracted RNA was then used for reverse transcription to synthesize cDNA using the miScript Reverse Transcription kit (Qiagen GmbH) and a Roche Lightcycler 480 Real Time PCR system (Roche Diagnostics, Switzerland).

### Real-time quantitative polymerase chain reaction (RT-qPCR)

After successfully extracting RNA and synthesizing cDNA as described above, RT-qPCR was performed to detect the relative expression levels of miR-501-3p in cell lines. The Roche Lightcycler 480 Real Time PCR system was used. The miScript Reverse Transcription kit (Qiagen GmbH, Germany) was used to detect mRNA levels. Reaction conditions were as follows: 95°C for 15 min, 40 cycles of 94°C for 15 s, 55°C for 30 s, and 70°C for 30 s. Calculations were performed using the 2-ΔΔCq method. The primer sequences can be seen in Table 1.

### CCK-8 assay

The CCK-8 assay was used to evaluate proliferation capacity of RCC cells. RCC cells were cultured in 96-well plates and divided into four groups. Subsequently, the four groups of cells were transfected with miR-501-3p mimics, inhibitors, and corresponding negative controls (nc). Concentration of miRNA mimics and inhibitors were both 31.15 nmol/L. After 0, 24, 48, and 72 h following transfection, 10 µL of CCK-8 reagent (Beyotime Institute of Biotechnology, China) was added to each well of cells and incubated for 30 min in the incubator at 37°C. Finally, the absorbance of each well was measured using a wavelength of 490 nm in the ELISA microplate reader (Bio Rad Laboratories, Inc., USA). The experiment was repeated three times.

### MTT assay

The 96-well plates were used for this assay. RCC cells from ACHN and 786-O were seeded and incubated in 96-well plates (5×10^3^ cells/well) for 24 h. Subsequently, each group of cells was transfected with miR-501-3p mimic, inhibitor, mimic nc, or inhibitor nc and incubated for 4 days. After that, in order to detect the absorbance to evaluate cell viability, about 20 µL MTT solution (5 mg/mL; Sigma-Aldrich, USA) was added into each well, and the medium was replaced with 100 µL dimethylsulfoxide (DMSO, Sigma-Aldrich, China) 4 h later. After 15 min of shaking in the reciprocating decolorization shaking table (TSB-108, Qilinbeier, China), absorbance was captured with the application of the ELISA microplate reader (Bio-Rad) at a 490 nm wavelength.

### Wound healing assay

ACHN and 786-O cells were seeded and cultured in 6-well plates and then transfected with miR-501-3p siRNA 24 h later. Concentration of miRNA mimics and inhibitors were both 45.25 nmol/L. After transfection, a straight and well-proportioned scratch was performed with a sterile 200-µL pipette tip on the surface of each well. Before capturing the images, loose cells were removed by washing with phosphate-buffered saline (PBS; Gibco; Thermo Fisher Scientific, Inc.). About 0 and 12 h after scratching, photos were obtained using a digital camera system (Olympus Corporation, Japan). The experiment was repeated three times.

### Transwell assay

Transwell chamber inserts (BD Biosciences, USA) containing Matrigel were used for invasion assays, and inserts without Matrigel were used for migration assays. For both assays, about 2×10^4^ of RCC cells, transfected with siRNAs 24 h before, were added to the upper chambers and diluted in 200 μL in serum-free DMEM (for ACHN) or 1640 (for 786-O) medium. Concentration of miRNA mimics and inhibitors were both 33.22 nmol/L. In addition, the bottom of the inserts was infused with 500 μL of DMEM (for ACHN) or 1640 (for 786-O) medium containing 10% FBS. After 24-h incubation, cells were fixed using paraformaldehyde and stained with crystal violet. Images were then captured using the Leica DMIRB (Germany) inverted microscope (magnification,×200). The experiment was repeated three times.

### Flow cytometry assay

ACHN and 786-O cells transfected with a miR-501-3p mimic, inhibitor, mimic nc, and inhibitor nc were cultured for 48 h at 37°C with 5% CO_2_. Cells were then collected, washed (cold PBS), and resuspended (100 μL 1× binding buffer). Next, about 5 μL of Annexin V-fluorescein isothiocyanate and 5 μL of propidium iodide (Invitrogen; Thermo Fisher Scientific, Inc.) were added to each cell resuspension solution. After 15 min in the dark, each resuspension was mixed with 400 μL of 1× binding buffer and was subjected to flow cytometry (EPICS, Xl-4; Beckman Coulter, Inc., USA) to detect early apoptosis rates. The experiment was repeated three times.

### Bioinformatics analysis and target gene prediction

miRDB (http://mirdb.org/index.html) is an online microRNA target prediction database. The potential target genes of miR-501-3p were predicted by miRDB and subsequently uploaded to the David database (https://david.ncifcrf.gov/home.jsp) for Gene Ontology (GO) function annotation and Kyoto Encyclopedia of Genes and Genomes (KEGG) pathway enrichment analysis. Then, based on the condition of high expression, |log2FC|> 1, and q-value <0.01, differentially expressed genes (DEGs) in RCC were obtained from The Cancer Genome Atlas (TCGA) database (https://www.cancergenome.nih.gov) with the LIMMA method. Subsequently, by cross-matching the target genes predicted by the miRDB database with the differentially expressed genes up-regulated in renal cancer from TCGA and drawing a Venn diagram, intersecting target genes could be obtained. Finally, in order to further verify the expression level and prognosis prediction value of the 13 DEGs in RCC, GEPIA (http://gepia.cancer-pku.cn/index.html) was used for expression detection and survival analysis. P<0.05 was considered statistically significant.

### Western blots

Western blot was used to identify the expression level of SPC24 in renal cancer cells. First, lysis buffer was added to the transfected renal cancer cells ACHN and 786-O for protein extraction. The polyacrylamide gel (SDS-PAGE) was prepared and added to the electrophoresis tank to separate 50 μg of protein by electrophoresis until the appearance of bromophenol blue. Then, the isolated protein was transferred with PVDF, and the PVDF membrane was washed with TBST solution and sealed with 5% skim milk. Finally, after incubation with primary antibody SPC24 (1:1000, Catalog: A16601, ABclonal, China), β-actin (1:1000, Catalog: ab8227, Abcam, China), and secondary antibody (goat anti-rabbit antibody, 1:10000, Jackson ImmunoResearch, USA), luminescence detection was performed. Immunoblot areas were evaluated using ImageJ (USA). The experiment was repeated three times.

### Dual-luciferase reporter assay

PmirGLO vectors (GenePharma, China) containing miR-501-3p sequences with wild-type (WT) or mutant (MUT) binding locations for SPC24 (SPC24-WT, SPC24-MUT) were adopted. 293T cells were co-transfected with miR-501-3p mimics or mimics nc and the luciferase vectors. Detection of luciferase activity was made with a Dual Luciferase Reporter Gene Assay Kit (China).

### Statistical analysis

Each experiment was repeated at least three times. Statistical significance was confirmed by the Student's *t*-test. The expression levels of miR-501-3p between RCC cells (ACHN, 786-O) and 293T cells were compared using paired *t*-test. All analyses were performed using SPSS 19.0 statistical software package (IBM SPSS, USA), and differences were considered statistically significant when P<0.05.

## Results

### MiR-501-3p was down-regulated in RCC cells

**Figure 1:**
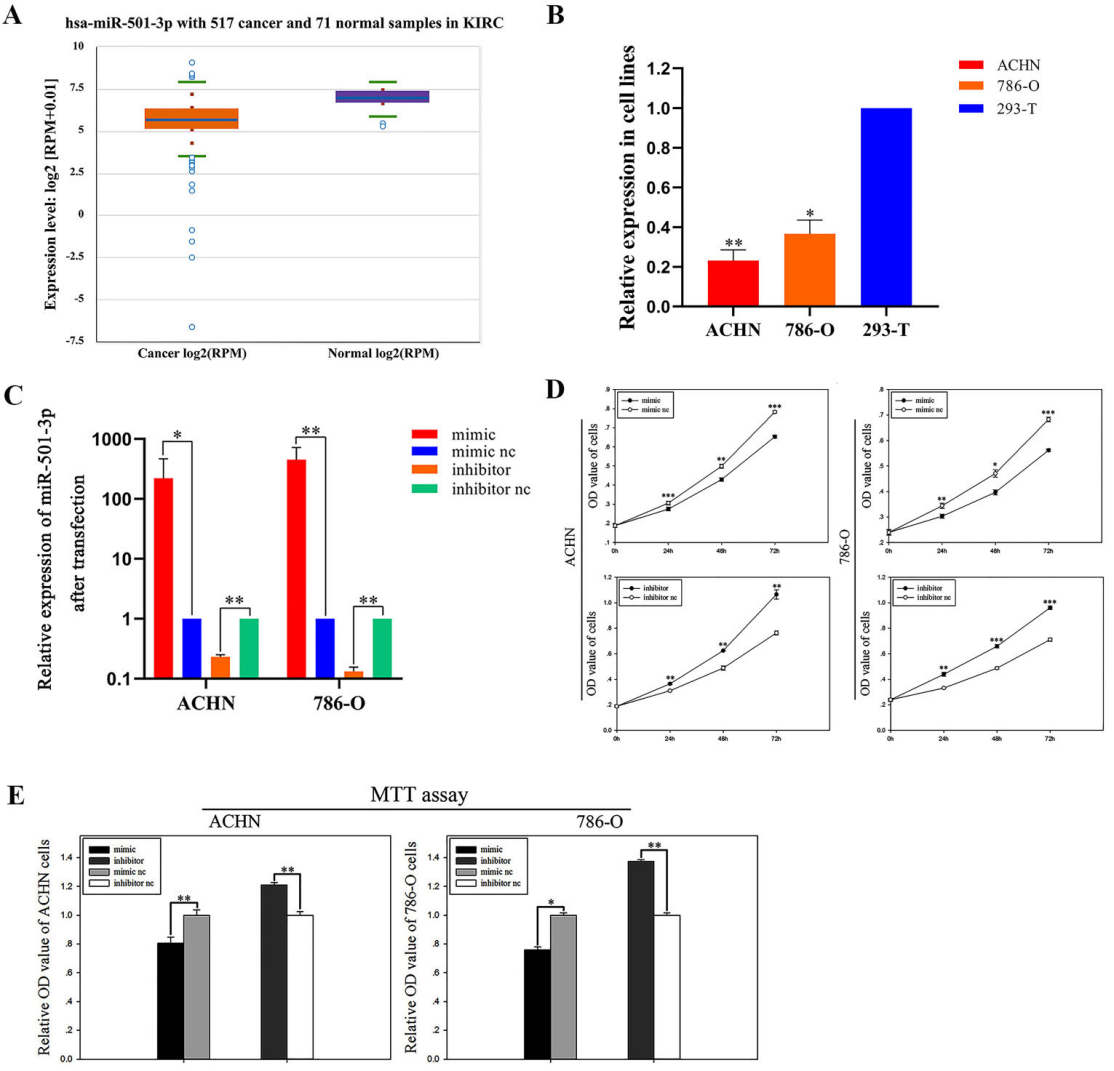
Expression levels of miR-501-3p. **A**, The StarBase V3.0 database showed that mir-501-3p was down-regulated in KIRC (kidney renal clear cell carcinoma) compared with normal tissues. **B**, The relative expression of miR-501-3p in renal cell carcinoma (RCC) cell lines. **C**, The expression of miR-501-3p after transfection in ACHN and 786-O cells. **D**, CCK-8 assay results. **E**, MTT assay results. Data are reported as mean and SD. *P<0.05, **P<0.01, ***P<0.001 (Student's *t*-test). nc: negative control; OD: optical density (absorbance).

The StarBase v3.0 database was used to analyze the TCGA data and the results revealed that miR-501-3p was down-regulated in KIRC (kidney renal clear cell carcinoma) (Figure 1A). Therefore, RT-qPCR was used to detect miR-501-3p expression levels in cell lines including ACHN, 786-O, and 293-T. Compared with 293-T cells, miR-501-3p expression levels were lower in RCC cell lines as shown in Figure 1B (ACHN, 0.23±0.08, P=0.00707; 786-O, 0.36±0.11, P=0.0121; 293T, 1.000±0.05).

### Transfection efficiency

Transfection efficiency of miR-501-3p siRNA was detected using RT-qPCR. Compared with the mimic nc, the expression levels of miR-501-3p increased by 145.68% in ACHN cells transfected with miR-501-3p mimic, and 368.79% in 786-O cells (Figure 1C). Also, compared with the inhibitor nc group, the expression levels of miR-501-3p decreased by 76.89% in ACHN cells transfected with inhibitor, and 86.88% in 786-O cells (Figure 1C).

### MiR-501-3p inhibited RCC cell proliferation

The CCK-8 assay was used to explore the role of miR-501-3p in RCC cell proliferation. Compared with the corresponding negative control, there were 10.25% (24 h, P<0.001), 14.00% (48 h, P<0.01), and 16.51% (72 h, P<0.001) decreases in ACHN cells transfected with miR-501-3p mimics, while there were 17.40% (24 h, P<0.01), 29.99% (48 h, P<0.01), and 39.53% (72 h, P<0.01) rises in the inhibitor group (Figure 1D). The same conclusion was found in 786-O cells, where there were 11.95% (24 h, P<0.01), 15.73% (48 h, P<0.05), and 17.70% (72 h, P<0.001) decreases present in cells transfected with a mimic and there were 32.50% (24 h, P<0.01), 35.19% (48 h, P<0.001), and 35.22% (72 h, P<0.001) increases in the inhibitor group (Figure 1D). Briefly, results revealed that miR-501-3p suppressed the growth of both ACHN and 786-O cells.

### MiR-501-3p inhibited RCC cell viability

In order to detect cell viability of ACHN and 786-O cells transfected with miR-501-3p siRNA, the MTT viability assay was performed. As shown in Figure 1E, cells with high expression of miR-501-3p had a lower survival rate, while low expression of miR-501-3p was associated with higher survival. Compared with the corresponding negative control, ACHN cells and 786-O cells, transfected with mimic, were decreased by 19.42% (P<0.01) and 24.08% (P<0.05), respectively, while in cells transfected with inhibitor, survival was increased by 20.86% (P<0.01) in ACHN cells and 37.23% (P<0.01) in 786-O cells.

### MiR-501-3p inhibited RCC cell migration

**Figure 2:**
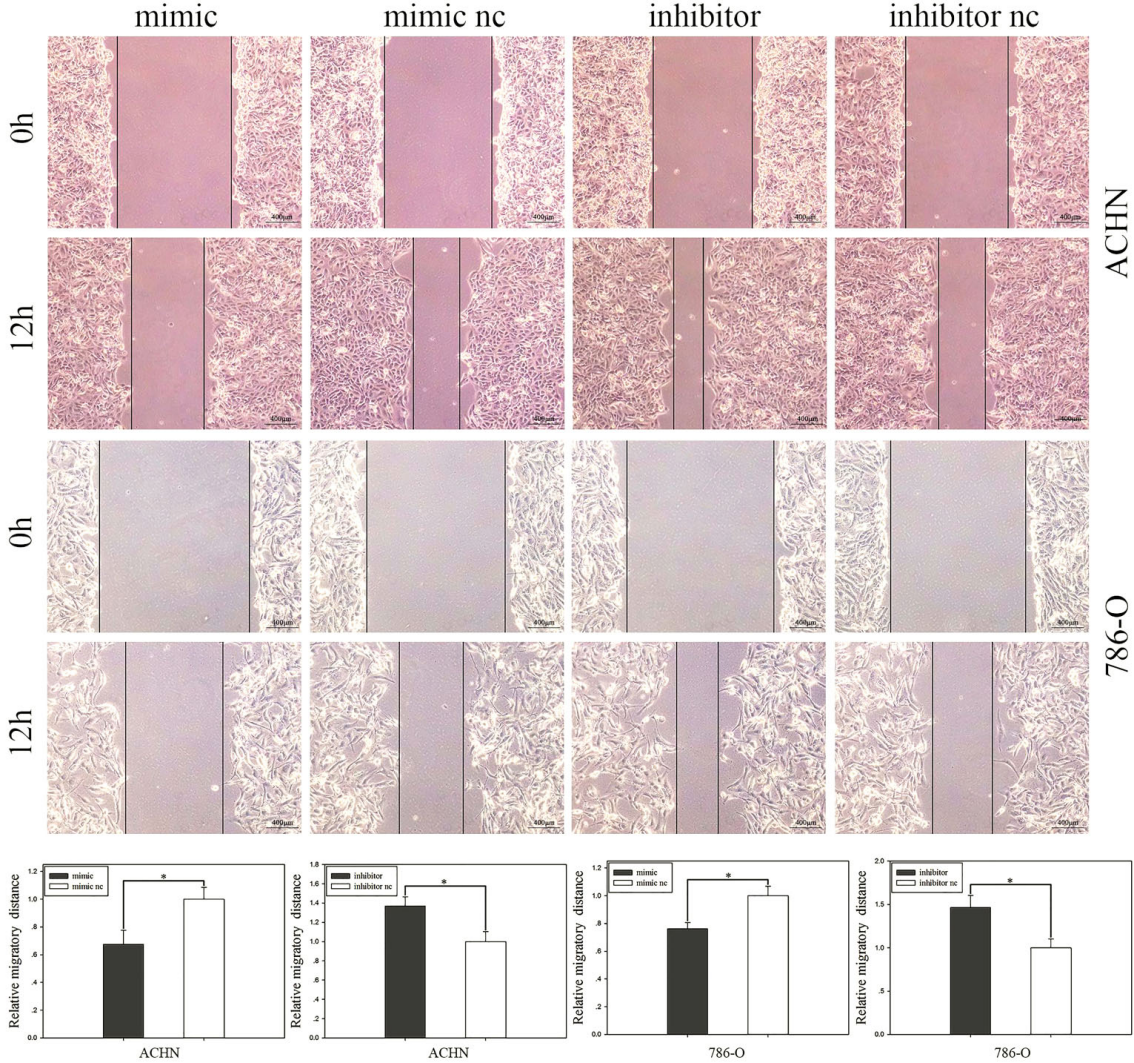
Wound healing assay. Images of cell migration and cell migratory distances after transfection with miR-501-3p mimic or mimic nc and inhibitor or inhibitor nc (upper panels, scale bar 400 μm). Data are reported as mean and SD. *P<0.05 (Student's *t*-test). nc: negative control.

To evaluate cell migration, the wound healing assay was performed. Compared with the corresponding control group, migration of ACHN cells decreased by 32.48% (P<0.05) in the mimic group and increased by 36.82% (P<0.05) in the inhibitor group (Figure 2). As for the 786-O group, compared with the negative control, migration decreased by 23.78% (P<0.05) in cells transfected with the mimic and increased by 46.36% (P<0.05) in cells transfected with the inhibitor (Figure 2).

**Figure 3:**
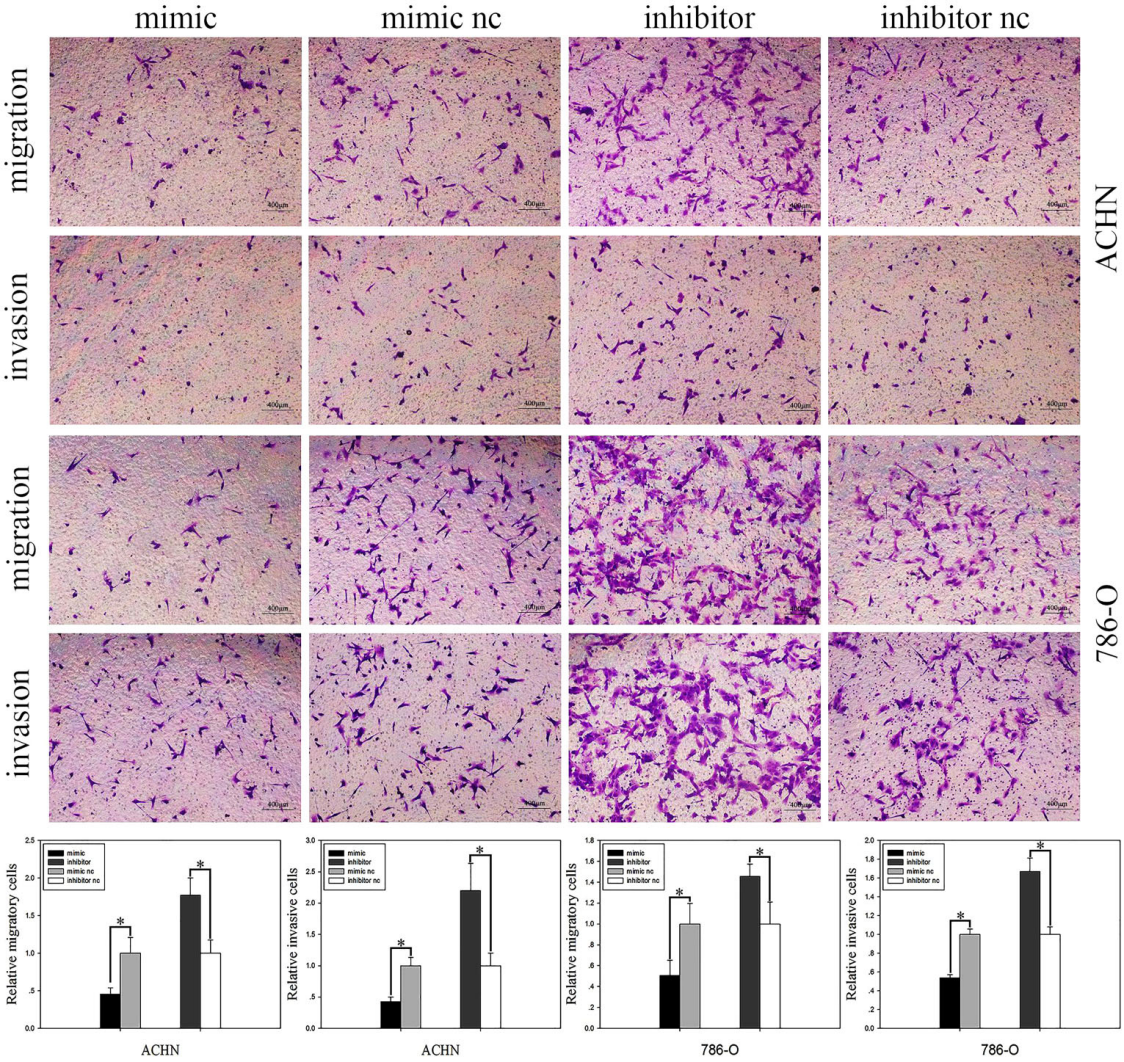
Transwell assay. Images of cell migration and invasion after transfection with miR-501-3p mimic or mimic nc and inhibitor or inhibitor nc (upper panels, scale bar 400 μm). MiR-501-3p inhibited migration and invasion abilities of cells. Data are reported as mean and SD. *P<0.05 (Student's *t*-test). nc: negative control.

In addition, the transwell assay was also used to analyze cell migration. In the ACHN groups, cell migration was reduced by 54.49% in mimic cells (P<0.05) and increased by 77.08% in inhibited cells (P<0.05) (Figure 3). As for 786-O (Figure 3), the number of migrated cells was reduced by 49.43% in mimic cells (P<0.05) and increased by 57.13% in inhibited cells (P<0.05).

### MiR-501-3p inhibited RCC cell invasion

The transwell assay showed that, compared to the control group, the invasive rate was reduced by 57.64% (P<0.05) in ACHN cells transfected with mimic and increased by 119.65% (P<0.05) in the inhibited group (Figure 3). Similarly, there was a 46.28% (P<0.05) reduction of 786-O cell invasion in the mimic group and a 67.03% (P<0.05) increase in inhibited cells compared with the corresponding control group (Figure 3).

### MiR-501-3p promoted RCC cell apoptosis

**Figure 4:**
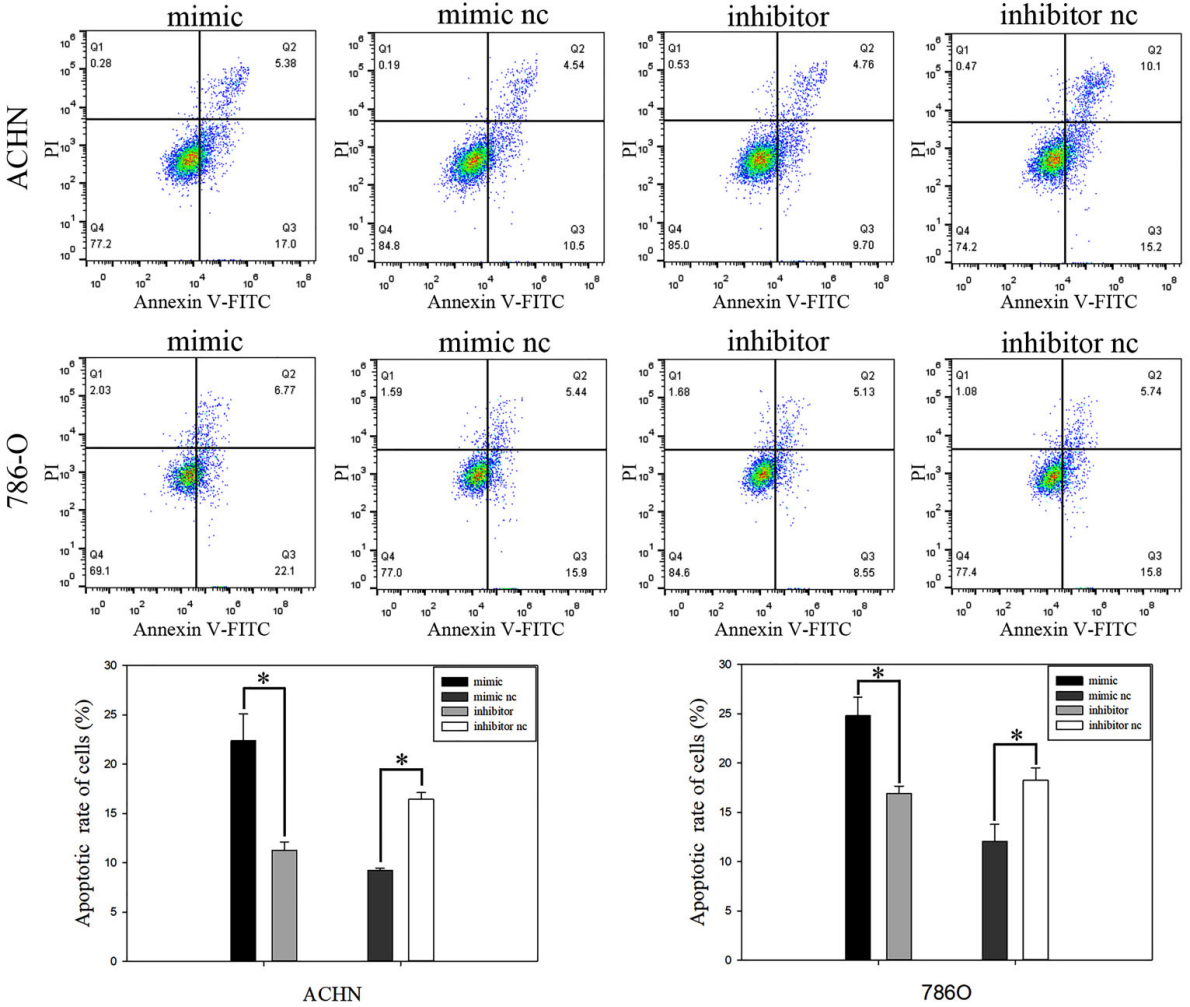
Flow cytometry results of renal cell carcinoma cell lines (upper panels). Cell apoptosis rate (%) of cell lines transfected with miR-501-3p mimic or mimic nc and inhibitor or inhibitor nc (lower panels). Data are reported as mean and SD. *P<0.05 (Student's *t*-test). nc: negative control.

Flow cytometry was used to determine apoptosis of RCC cells. Results showed that miR-501-3p promoted apoptosis of RCC cells (Figure 4). In ACHN cells transfected with a miR-501-3p mimic, the early apoptosis rate was 22.4% (P<0.05.), which was higher than the negative control group. In 786-O cells, early apoptosis increased by 24.8% (P<0.01). In contrast, cells transfected with miR-501-3p inhibitors showed a lower apoptosis rate compared with the control group. The early apoptotic rates were 9.2% (AHCN; P<0.05) and 12.0% (786-O; P<0.01), which were lower than their corresponding negative controls.

### GO and KEGG

**Figure 5:**
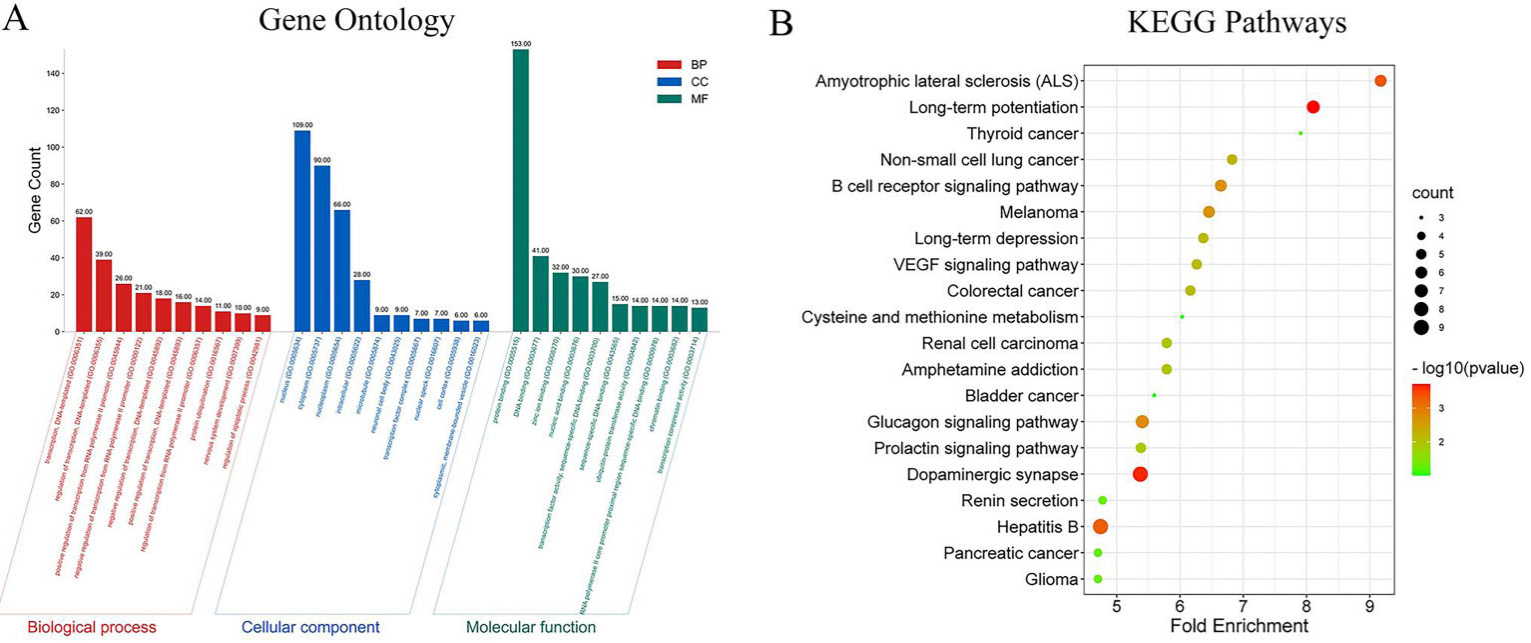
**A**, Functional enrichment analysis of miR-501-3p candidate target genes predicted from the miRDB database (BP: biological process, CC: cellular component, MF: molecular function). **B**, KEGG pathway enrichment analyses of the potential target genes of miR-501-3p from miRDB are shown.

Through miRDB prediction, we obtained 289 candidate DEGs of mir-501-3p. To identify the potential mechanisms by which predicted target genes participate in cell activities, we annotated the GO function using the David database (Figure 5A). The top 10 biological processes (BPs) in terms of gene count were transcription, regulation of transcription, positive regulation of transcription from RNA polymerase II promoter, negative regulation of transcription from RNA polymerase II promoter, negative regulation of transcription, DNA-templated positive regulation of transcription, DNA-templated regulation of transcription from RNA polymerase II promoter, protein ubiquitination, nervous system development, and regulation of the apoptotic process. The top 10 cellular components (CCs) in terms of gene count were nucleus, cytoplasm, nucleoplasm, intracellular, microtubule, neuronal cell body, transcription factor complex, nuclear speck, cell cortex, cytoplasmic, and membrane-bounded vesicle. The top 10 molecular functions (MFs) in terms of gene count were protein binding, DNA binding, zinc ion binding, nucleic acid binding, transcription factor activity, sequence-specific DNA binding, ubiquitin-protein transferase activity, RNA polymerase II core promoter proximal region sequence-specific DNA binding, chromatin binding, and transcription corepressor activity. KEGG pathway enrichment analysis was also performed (Figure 5B). According to the fold enrichment score, the top 20 pathways were Amyotrophic lateral sclerosis (ALS), Long-term potentiation, Thyroid cancer, Non-small cell lung cancer, B cell receptor signaling pathway, Melanoma, Long-term depression, VEGF signaling pathway, Colorectal cancer, Cysteine and methionine metabolism, Renal cell carcinoma, Amphetamine addiction, Bladder cancer, Glucagon signaling pathway, Prolactin signaling pathway, Dopaminergic synapse, Renin secretion, and Hepatitis B, Pancreatic cancer, Glioma.

### Bioinformatics analysis and target gene prediction

**Figure 6:**
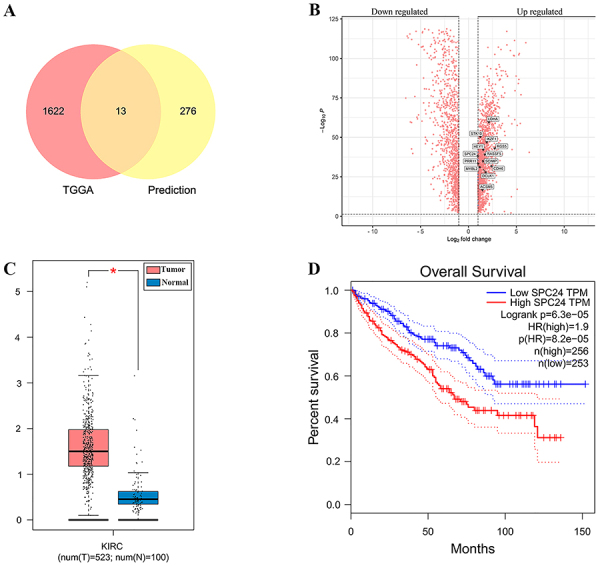
Potential target genes of miR-501-3p were predicted. **A**, Venn diagram showed 13 overlapping genes, the intersection of 289 potential target genes predicted by miRDB and 1652 up-regulated genes in renal cancer from TCGA database. **B**, Differentially expressed genes in renal cell carcinoma (TCGA database). **C**, SPC24 was highly expressed in kidney renal clear cell carcinoma (KIRC). Data are reported as median and interquartile range. *P<0.05 (Mann-Whitney test). **D**, The survival analysis showed that SPC24 was associated with poor prognosis in KIRC. HR: hazard ratio.

With the extraction and screening of TCGA data, 2859 differentially expressed target genes in renal cancer were obtained by cross-matching the target genes predicted by the miRDB database with the up-regulated DEGs in renal cancer from TCGA, and drawing a Venn diagram. We obtained 13 intersecting target genes, and the results are shown in Figure 6A. The 13 candidate target genes are marked in Figure 6B.

In order to further verify the expression level and prognosis prediction value of the 13 DEGs in RCC, GEPIA (http://gepia.cancer-pku.cn/index.html) was used for expression detection and survival analysis. The results showed that SPC24 was up-regulated in RCC (Figure 6C), and the up-regulation was negatively correlated with the expression of miR-501-3p. Furthermore, up-regulation of SPC24 was associated with poor overall survival in RCC patients (Figure 6D). Based on the fact that miRNAs act to negatively regulate target gene expression by binding to the 3′-UTR of mRNAs, we speculated that miR-501-3p exerted its tumor suppressor and favorable prognostic effects by targeting SPC24.

### SPC24 was the potential target gene of miR-501-3p

**Figure 7:**
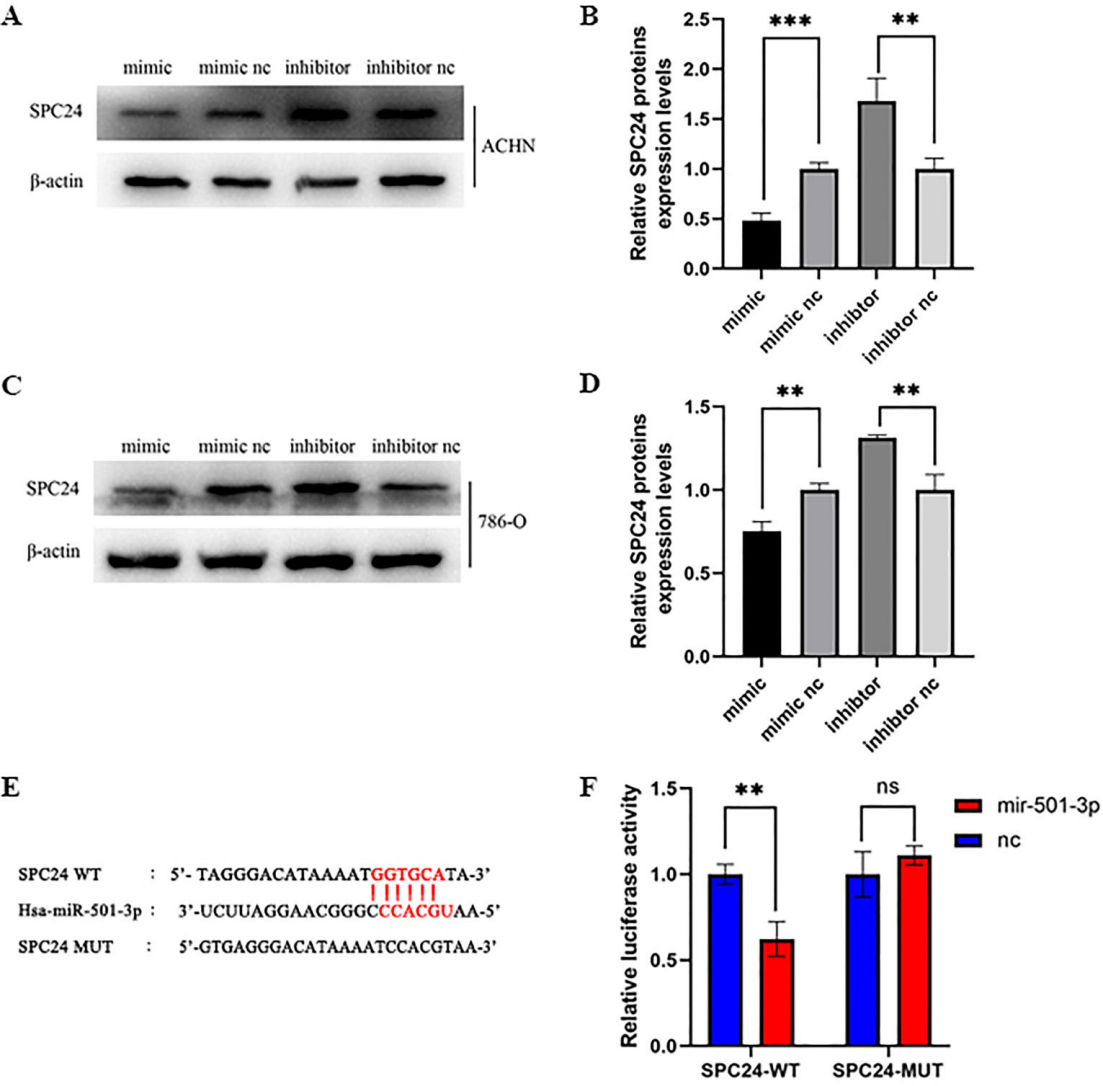
SPC24 was directly targeted by mir-501-3p. **A**-**D**, Western blot was performed to assess the expression of SPC24 in renal cancer cells transfected with miR-501-3p mimic, inhibitor, and corresponding negative control (nc) groups. **E**, The target sequence of miR-501-3p in the wild type (WT) and mutated (MUT) SPC24. **F**, The dual-luciferase reporter assay was performed, and miR-501-3p weakened the luciferase activity of SPC24-WT and had no substantial effect on SPC24-MUT. Data are reported as mean and SD. **P<0.05, ***P<0.01 (Student's *t*-test).

As shown in Figure 7, the putative target sequence of miR-501-3p is in the 3′UTR of SPC24. In order to verify the prediction of the target genes, western blot was performed. The results showed that the expression of SPC24 in renal cancer cells transfected with miR-501-3p mimic was decreased compared with the control groups (Figure 7A-D). In contrast, we observed that the expression of SPC24 was increased in ACHN or 786-O cells transfected with the miR-501-3p inhibitor compared to the control groups (Figure 7A-D). Similarly, dual-luciferase reporter assay results demonstrated that miR-501-3p weakened the luciferase activity of SPC24-WT, but it had no substantial effect on SPC24-MUT (Figure 7E and F). The experiment was repeated 3 times with statistical significance (P<0.05). To sum up, SPC24 was the potential target gene of miR-501-3p in renal cell carcinoma, and miR-501-3p may affect the biology of renal cancer cells by regulating the expression of SPC24.

## Discussion

In China, RCC is the most common urological malignancy seriously threatening health and life (13). Due to poor effects of radiotherapy and chemotherapy for RCC treatment, surgical resection is still an important and effective method used for treatment (14). However, due to the difficulty of early diagnosis, many RCC patients are diagnosed at an advanced stage and there is no opportunity for radical surgery (2). In addition, due to the lack of tumor-specific markers, the monitoring of renal cancer recurrence still relies on imaging studies, and it is difficult to predict the prognosis of patients with renal cancer (15,16). Since there is a lack of indicators for monitoring recurrence and prognosis, many kidney cancer patients do not have simple, effective, and beneficial disease management after surgery, leading to a decline in life quality and shortened OS (3,17). Therefore, this study was dedicated to revealing tumor markers for renal cancer, which could aid in early diagnosis, intervention, and postoperative disease management of RCC.

In recent years, many scholars have performed research investigating microRNAs. MicroRNAs indirectly regulate gene expression by regulating mRNA levels after gene transcription to influence the biological behavior of tumor cells (18-20). For instance, Tupone et al. (20) uncovered that miR-378a-5p is increased in metastatic melanoma specimens and demonstrated that overexpression of miR-378a-5p enhanced migration, cell invasion, and proliferation in melanoma cells. In addition, Al-Abdallah et al. (21) found that miR-146b-5p affects papillary thyroid carcinoma occurrence by regulating the stress-activated protein kinase pathway and expression of stannin-1. As for RCC, there is much research on miRNAs, especially about roles of miRNA in RCC prognosis prediction. For example, Kurozumi et al. (22) found that the expression of the miR-200 family miRNAs may be related to kidney cancer by literature reviews and computer analyses. Petrozza et al. (23) found that the expression of miR-210-3p was up-regulated in the tumor tissues and urine samples of 21 clear cell renal cell carcinoma (ccRCC) patients by RT-qPCR. The research results revealed the role of miR-210-3p as a prognostic marker of ccRCC. Lokeshwar et al. (24) performed RT-qPCR and discovered that the miR combinations (miR-21+194; miR-21+142-5p+194) may be the potential markers for early diagnosis and prognosis prediction in RCC. Morris and Latif (25) discussed the effects of DNA methylation, microRNA dysregulation, and histone modification enzyme mutations on renal cancers through a literature review. Saleeb et al. (26) found that patients with positive expression of miR-141, miR-200b, and miR-200c had a higher survival rate through RT-qPCR, clinicopathological data comparison, and bioinformatics analysis. Therefore, they proposed that miR-141, miR-200b, and miR-200c may be prognostic markers of ccRCC. miR-501-3p was found to be related to the occurrence and development of many tumors. For example, Luo et al. (8) reported that mRNA-501-3p suppressed metastasis and progression of hepatocellular carcinoma by targeting LIN7A. Sanches et al. (27) found that miR-501 was increased in cervical cancer and promoted cell proliferation, migration, and invasion by targeting CYLD lysine 63 deubiquitinase (CYLD).

To this day, there is a lack of research investigating the relationship between miR-501-3p and SPC24 in kidney cancer. According to data extracted from the TCGA database, miR-501-3p was found to be down-regulated in RCC tissues. Thus, we speculated that miR-501-3p may be a potential tumor suppressor gene. To confirm this observation, RT-qPCR was used to test the miR-501-3p expression levels in RCC cell lines. Compared with normal cells, expression of miR-501-3p in RCC was lower. RT-qPCR results revealed a similar conclusion to TCGA data. Subsequently, phenotypic experiments in RCC cell lines were performed. In the CCK-8 experiment, RCC cells transfected with a miR-501-3p mimic showed a decrease in proliferation, while cells transfected with the inhibitor showed increased proliferation ability. In the wound healing assay, compared with the corresponding control group, migration length of ACHN and 786-O cells decreased with high expression of miR-501-3p. In transwell experiments, it increased miR-501-3p and hindered cell migration and invasion. In addition, flow cytometry experiments revealed that miR-501-3p expression levels were positively correlated with early apoptosis.

In brief, using functional assays such as CCK-8, migration, invasion, and flow cytometry, we found that miR-501-3p negatively regulated the activity and behavior of RCC, indicating that it may serve as a potential suppressor gene. In order to clarify the possible mechanism of miR-501-3p tumor suppressor effect in RCC, several databases were applied, such as TCGA database, miRDB database, David database, and GEPIA. The results showed that SPC24 was up-regulated in RCC, and the up-regulation was negatively correlated with the expression of miR-501-3p. In addition, up-regulation of SPC24 was associated with poor overall survival in RCC patients. Therefore, we speculated that miR-501-3p exerted its tumor suppressor and favorable prognostic effects by targeting SPC24. Finally, results of western blot and dual-luciferase reporter assay showed that the expression level of SPC24 could be suppressed by miR-501-3p in renal cell cancer cells, and SPC24 was directly targeted by mir-501-3p.

However, there were limitations in this study. We only found the miR-501-3p target SPC24, but did not explore the mechanism and function of SPC24 in renal cancer. The mechanism of miR-501-3p/SPC24 axis effects on the biological behavior of renal cancer cells is still unclear. Due to limitations in experimental facilities, we did not conduct animal experiments, making it difficult to fully reveal the pathogenesis of mir-501-3p *in vivo*.

In addition, our facility is deficient in clinical research, and therefore the value of early diagnosis, recurrence monitoring after surgery, and prediction of RCC prognosis could not be assessed.

The clinical applications of miRNA include early diagnosis, postoperative recurrence monitoring, and prognosis prediction, which have great significance for the management of RCC. RCC is not sensitive to radiotherapy and chemotherapy (28), and surgery is still the most effective treatment. The benefit of surgery depends on tumor size and invasion degree. When the tumor is smaller and has a low invasion degree, surgical treatment has the best effect. Therefore, early diagnosis is of great significance for the treatment of RCC. On the other hand, RCC has a high recurrence rate after surgery (29), thus postoperative monitoring and prognosis prediction are key to formulate the treatment plan after surgery, which can maximize survival.

Relevant studies have shown that there is a good correlation between the expression levels of miRNAs in plasma and in primary tumor tissues (30,31). Kim et al. (32) found through TCGA database and RT qPCR analysis validation that the miRNA combination (miR-9, miR-16, miR-21, and miR-429) is highly expressed in early breast cancer, which shows sensitivity and specificity in early diagnosis of breast cancer. He et al. (33) followed prostate surgery patients for two years and found that miR-148a-3p and miR-485-5p may be a new miRNA combination of recurrence diagnostic markers. Teixeira et al. (34) analyzed the relative expression of miR-221 in plasma samples of RCC patients and found that patients with higher levels of miR-221 expression had significantly reduced OS, and miR-221 expression had great predictive ability for RCC prognosis prediction.

In summary, we believe that miR-501-3p has the potential for clinical application in early diagnosis, recurrence detection, and prognosis prediction. Future research on the above aspects should explore the mechanism of miR-501/SPC24 in renal cell carcinoma.
